# The value of elbow arthroscopy in diagnosing and treatment of radial head fractures

**DOI:** 10.1186/s12891-019-2726-6

**Published:** 2019-07-27

**Authors:** Florian Haasters, Tobias Helfen, Wolfgang Böcker, Hermann O. Mayr, Wolf Christian Prall, Andreas Lenich

**Affiliations:** 1 Hospital of General, Trauma and Reconstructive Surgery, University of Munich (LMU), Nussbaumstr. 20, 80336 Munich, Germany; 2 Department of Knee, Hip and Shoulder Surgery, Schön Klinik Munich-Harlaching, Academic Teaching Hospital of the Paracelsus Private Medical University Salzburg, Strubergasse 21, 5020 Salzburg, Austria; 3 Department of Orthopaedics and Traumatology, Freiburg University Hospital, Albert-Ludwigs-University of Freiburg, Hugstetterstrasse 55, 79106 Freiburg im Breisgau, Germany; 4 Department of Orthopedic Sports Medicine, University Hospital Rechts der Isar, Technical University Munich, Ismaninger Str. 22, 81675 Munich, Germany

**Keywords:** Radial head fracture, Arthroscopy, Associated injury, Elbow dislocation, Arthroscopic assisted fracture treatment

## Abstract

**Background:**

Surgical treatment of radial head fractures is increasingly performed arthroscopically. These fractures often feature concomitant injuries to the elbow joint, which may be under-diagnosed in the radiological examinations. Little is known about the diagnostic value of arthroscopy, the treatment options that arise from arthroscopically assisted fracture fixation and clinical results. We hypothesized that arthroscopy can detect additional concomitant injuries and simultaneously expands the therapeutic options. Therefore aim of this study was to compare arthroscopic and radiologic findings, to assess the distinct arthroscopic procedures and to follow up on the clinical outcomes.

**Methods:**

Twenty patients with radial head fractures were retrospectively included in two study centers. All patients underwent elbow arthroscopy due to at least one of the following suspected concomitant injuries: osteochondral lesions of the humeral capitellum, injuries of the collateral ligaments or loose joint bodies. Preoperative radiological findings were compared to arthroscopic findings. Afterwards, arthroscopic treatment options and clinical outcomes were assessed.

**Results:**

Arthroscopic findings led to revision of the classified fracture type in 70% (*p* = 0.001) when compared to preoperative conventional radiographs (CR) and in 9% (*p* = 0.598) when compared to computed tomography (CT) or magnetic resonance imaging (MRI). Diagnosis of loose bodies was missed in 60% (*p* < 0.001) of the CR and in 18% (*p* = 0.269) of the CT/MRI scans. Osteochondral lesions were not identified in 94% (p < 0.001) of the CR and in 27% (*p* = 0.17) of the CT/MRI scans. Percutaneous screw fixation was performed in 65% and partial radial head resection in 10%. Arthroscopy revealed elbow instability in 35%, leading to lateral collateral ligament reconstruction. After a mean follow up of 41.4 ± 3.4 months functional outcome was excellent in all cases (DASH-Score 0.6 ± 0.8; MEPI-Score 98.5 ± 2.4; OES-Score 47.3 ± 1.1).

**Conclusions:**

Elbow arthroscopy has a significant diagnostic value in radial head fractures when compared to standard radiological imaging. Although statistically not significant, arthroscopy also revealed concomitant injuries in patients that presented with an uneventful MRI/CT. Furthermore, all intraarticular findings could be treated arthroscopically allowing for excellent functional outcomes.

**Trial registration:**

Institutional Review Board University of Munich (LMU), Trial Number 507–14.

## Background

Management of radial head fractures is still discussed controversially, as there still is uncertainty and controversy about when surgery is needed as well as what type of surgical intervention is best. [1, 2] Most of the isolated (simple) fractures can be considered as stable and are suitable for a non-operative treatment. However, fractures associated with concomitant osseous or soft-tissue injury (complex fractures) require different treatment strategies to preserve and restore the integrity of the radiocapitellar joint and elbow stability [2, 3]. Multiple factors, such as fragment number, fragment displacement, articular impaction or radiocapitellar malalignment [4] as well as osteochondral lesions, loose joint bodies and elbow instability should guide surgical treatment [5]. In order to assess these factors, conventional radiographs (CR) in three planes are still the diagnostic standard [4, 6] while computed tomography (CT) and magnetic resonance imaging (MRI) are only occasionally applied to increase information about complex or ligamentous injury patterns [7].

Based on radiographic findings fractures are most widely classified according to the modified Mason classification [8, 9]. Type I fractures are defined as non-displaced or minimally displaced fractures that do not block motion. These fractures can be treated non-operatively [3, 4, 10]. Type II fractures are displaced fractures (> 2 mm) without comminution and with or without mechanical block of motion. The treatment recommendations are inconsistent since recent data revealed no differences in the functional outcome after surgical and non-operative treatment [2, 11]. Type III fractures are defined as displaced fractures involving the entire radial head that are deemed not repairable and should be either excised or replaced with a prosthesis [2, 9]. Johnston extended the classification system introducing radial head fractures associated with elbow dislocations (Type IV). [12] The accompanying elbow dislocation is likely change the prognosis in comparison to a similar fracture without dislocation. Following these classification systems, the question raises whether radiological examinations adequately capture all relevant injuries, or whether they are prone to miss injuries that play an important role for clinical outcome and treatment decision. Among these injuries, rotational block of motion, radial head fragments scattered into the posterior compartment, osteochondral lesions to the postero-lateral capitellum and postero-lateral instability may be underdiagnosed. In this context, elbow arthroscopy may represent a useful diagnostic tool complementing radiological findings and simultaneously allowing for minimally invasive treatment of the identified injuries [13, 14].

Elbow arthroscopy dramatically evolved within the last decades and became an integral part of diagnosing and an important assisting tool in treatment of a large variety of elbow injuries [13–19]. However, only a few case series reported on arthroscopically treated radial head fractures, such as screw osteosynthesis of Type II fractures or head resection after Type III fractures [20–22].

Therefore, aim of this study was to assess the significance of elbow arthroscopy in diagnosing and treatment of radial head fractures with associated injuries. Primary objective was to compare preoperative imaging to the arthroscopic findings. Secondary objectives were, weather all arthroscopic findings could be arthroscopically addressed, and the assessment of the mid-term functional outcomes. Our hypothesis was that elbow arthroscopy provides a more accurate fracture classification, a higher sensitivity for identification of associated intraarticular injuries and allows for simultaneous arthroscopic treatment of all lesions diagnosed.

## Methods

**Fig. 1:**
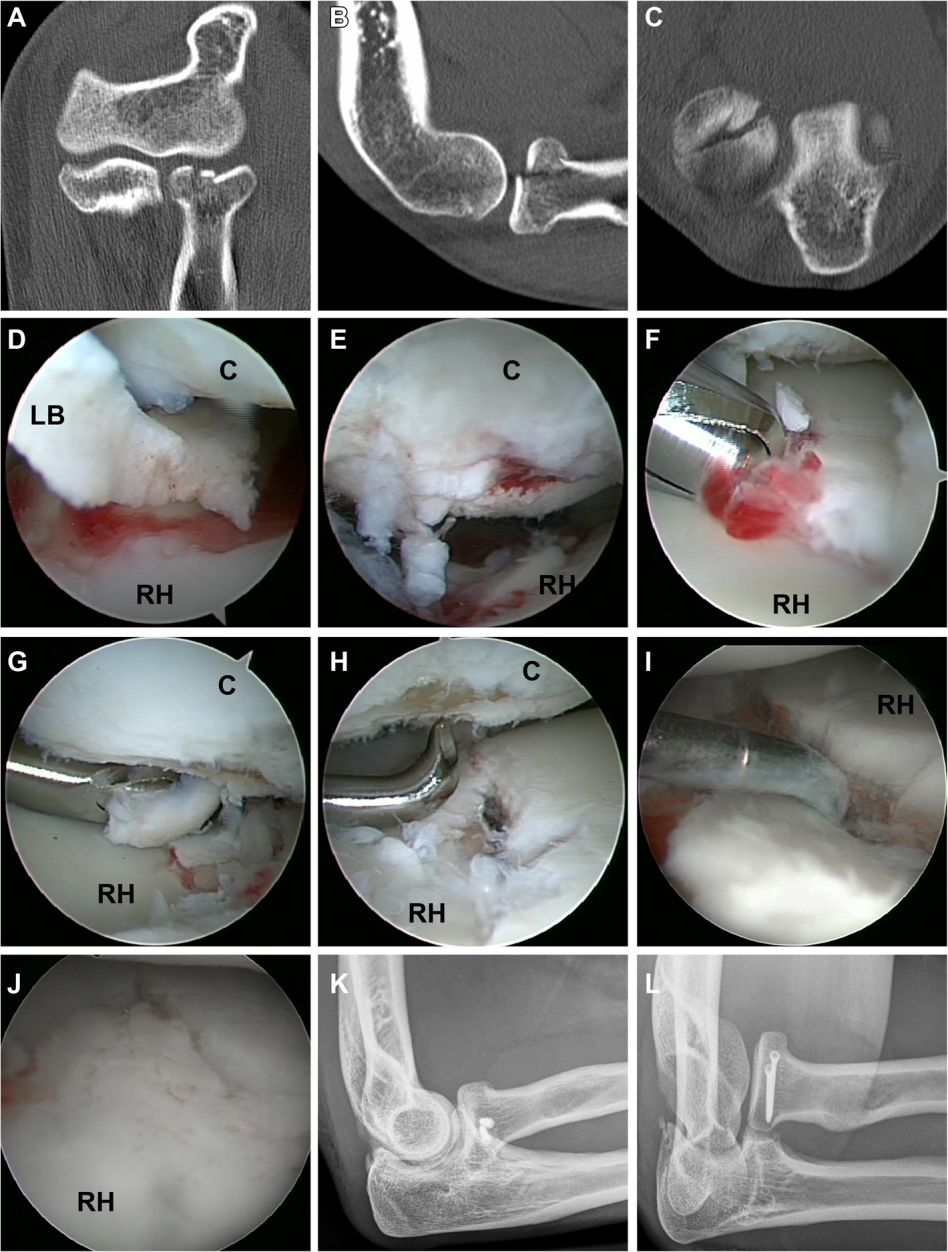
Mason Type II radial head fracture with traumatic capitellar chondral lesion und loose joint bodies. **a**-**c** CT scan showed a dislocation of 3 mm in coronal view. Loose bodies and capitellar injury were not identified (**d**) Arthroscopy revealed a large chondral loose body [LB] entrapped between the capitellum [C] and the radial head [RH]. **e** grade IV chondral lesion to the capitellum. **f** After removal of fracture hematoma, (**g**) chondroplastic and (**h**) microfracturing was performed at the capitellum humeri. **i** Fracture reduction was carried out with a sharp hook and (**j**) anatomic restoration of the radial head was achieved by screw osteosynthesis over the anterolateral portal. **k,l** Postoperative x-rays demonstrate anatomic reduction and correct screw placement

**Fig. 2:**
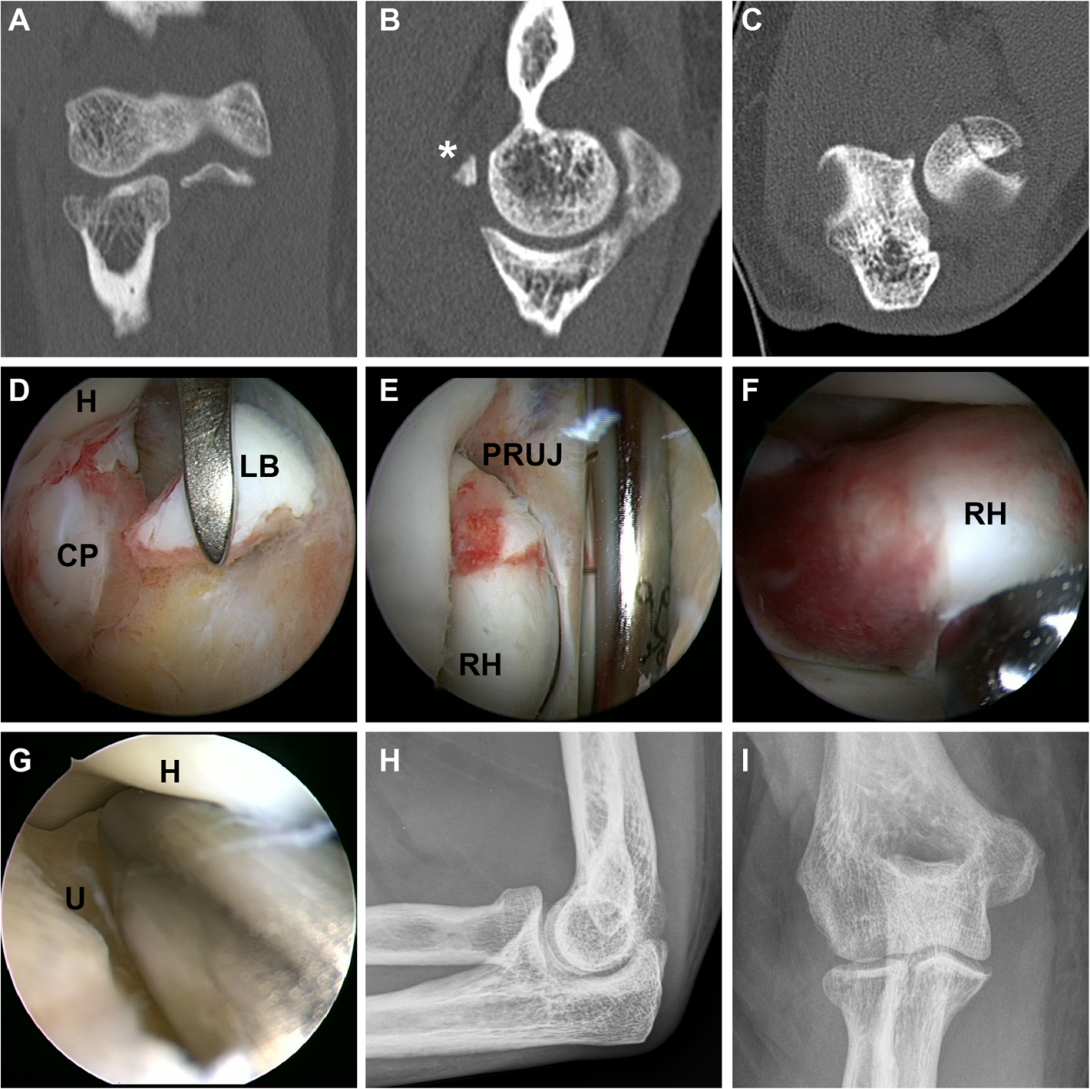
Mason Type I Fracture with loose joint body in the anterior compartment. **a**-**c** CT scan showed a mildly (< 2 mm) displaced radial head fracture without blocking of motion and an osteochondral fracture fragment in the anterior elbow compartment. **d** The loose joint body [LB] was arthroscopically removed after identification between humerus [H] and coronoid process [CP]. **e** Dynamic evaluation of unimpaired motion in the proximal radioulnar joint [PRUJ] was arthroscopically confirmed. **f** Exploration of the radial head in full supination. **g** Posterolateral rotational instability was ruled out with a modified “drive through test” with a switching stick from the soft spot portal between ulnar [U] and humerus [H]. **h, i** Postoperative x-rays demonstrated correct alignment of the elbow joint and complete removal of loose bodies

**Fig. 3:**
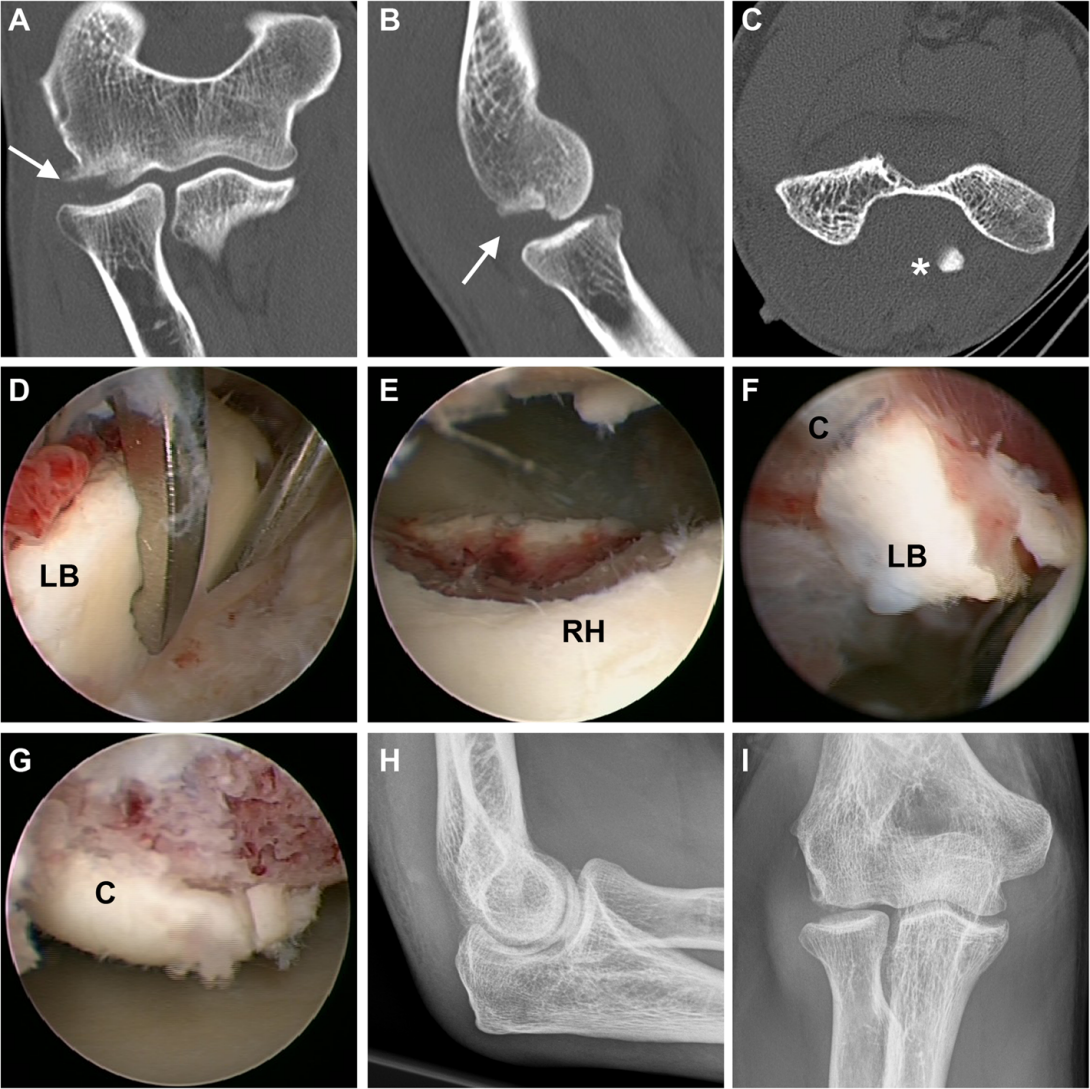
Radial head fracture classified as a type IV fracture due to an Osborne-Cotterill lesion [arrow] and displacement of the anterior rim fragment of the radial head into the fossa olecrani [*]. **a-c** CT scans. **d** Arthroscopic loose body [LB] removal. **e** Result after partial resection of unstable anterior radial head [RH] fragments. **f** A second loose body was found at the dorsal capitellum [C] near the (**g**) Osborne-Cotterill lesion. **h, i** Postoperative x-rays demonstrated correct alignment of the elbow joint and complete removal of loose bodies

In this retrospective case series we included all patients who underwent arthroscopically assisted surgical treatment following an acute (< 14 days) traumatic radial head fracture with associated injuries between May 2013 and May 2014. Inclusion criteria were any type of radial head fracture in patients > 18 years in combination of one of the following concomitant injuries: loose joint bodies, (osteo-)chondral lesions to the humeral capitellum, and injuries to the lateral or medial ligament complex (Figs. 1, 2 and 3). Diagnosis of the concomitant injury was performed according to radiological or clinical findings. CR in three planes was conducted in all cases. If any of the defined associated injuries was clearly diagnosed in plain radiographs, arthroscopy was performed without additional CT or MRI imaging. In case of insufficient visualization of the extend of dislocation, capitellar lesions, suspicion of loose joint bodies in CR a CT or MRI (if possible in full extension) was performed. Furthermore, in patients with an instability in the clinical examination or with a mechanical block of motion that could not be explained by CR findings, MRI or CT was conducted.

Exclusion criteria were open fractures, fractures deemed irreconstructable, radial neck fractures, accompanying fractures at other locations, terrible triad injuries, neuro-vascular injuries, neurological disorders or substance abuse that impaired postoperative compliance. Over the study period a total number of *n* = 59 patients have been treated by standard open reduction and internal fixation or radial head replacement.

Fracture classification was performed after CR, CT/MRI and during arthroscopy according to the Hotchkiss modified Mason classification with the Type VI extension according to Johnston.

Patients underwent general anesthesia and were placed in the lateral decubitus position. An examination under anesthesia (EUA) was performed under fluoroscopic imaging in order to assess elbow stability in full extension and 30° flexion. The arm was supported by means of a small arm holder allowing for a wide range of elbow motion. A tourniquet was inflated to 250 mmHg and the pump was set at 30 mmHg. Before portal placement, the joint was injected with 15 to 20 mL of saline solution through the soft spot. All arthroscopic procedures were performed by two senior surgeons. A standardized diagnostic evaluation for associated joint pathologies was carried out with a 4 mm 30° arthroscope in every case beginning from a posterolateral portal. A full-radius blade shaver was used through the transtricipital portal to remove hematoma and to allow for removal of loose bodies in the dorsal recessus (Fig. 3d). The arthroscope was then guided into the humeral radial joint thereby carefully evaluating the posterior and postero-lateral aspect of the capitellum (Fig. 3g). Through the soft spot portal the shaver blade was inserted to remove hematoma and loose bodies. The forearm was now rotated in pronation and supination in order to explore the radial head, to analyze the fracture pattern and to assess an eventual mechanical block of motion (Fig. 1). An anterolateral portal was then established to allow for visualization of the anterior elbow joint. An additional anteromedial portal was used if necessary. The “elbow drive-through test” (Fig. 2g) was performed in order to screen for posterolateral rotational instability (PLRI) as described previously [15, 16, 23].

Depending on the fracture pattern the portals used for visualization, fracture reduction and screw placement differed. However, in most cases the arthroscope was switched to the posterolateral portal while reduction and temporary fixation were performed with a sharp hook using the soft spot and anterolateral portal (Fig. 1). Subsequently, 2.0 mm screws (Medartis, Switzerland or Depuy-Synthes, USA) were placed via the anterolateral portal with the elbow flexed between 45° to 90° and in different extend of supination. Finally, fluoroscopic images were carried out in anteroposterior, lateral and oblique views. The arm was immobilized in a cast until the patient regained full consciousness and pain or swelling decreased. A pain-guided active-assisted physiotherapy was started at day one after surgery without weight bearing for 6 weeks.

Two senior surgeons performed evaluation of the preoperative radiological imaging independently. In case of different results, a consensus was found by discussion. Clinical results were assessed using the Disabilities of Arm, Shoulder and Hand Questionnaire (DASH), the Oxford Elbow Score (OES) and the Mayo Elbow Performance Index (MEPI) [24–26].

Pearson’s chi-squared test (χ [2]) was used to test for the association between categorical variables. The level of statistical significance was defined as *p* < 0.05.

All procedures were performed in accordance with the ethical standards of the institutional (votum no. 507–14) and national research committee and with the 1964 Helsinki declaration and its later amendments or comparable ethical standards. Informed written consent was obtained from all individual participants included in the study.

## Results

A total of 20 patients were included in this multicenter case-series. The mean age was 42.9 ± 10.9 years and 13 of 20 patients were male. The mean follow-up was 41.4 (±3.4, range 35.2–46.6) months (Table 1). All 20 patients received CR in three planes (anteroposterior, lateral and radiocapitellar view). In eight patients CT imaging and in three patients MRI scans were performed according to the above mentioned criteria (Table 2). Based on CR fracture were classified type I in 35%, type II in 35%, type III in 20% and type IV in 10%. Classification changed due to CT/MRI imaging in 7 of 11 (64%) cases (Table 3). In detail, two type I fractures changed to type II, two type I fractures changed to type IV, two type III fractures changed to type IV and one type II fractures changed to type IV (Table 2).

**Table 1:** Patient Data

Patient	1	2	3	4	5	6	7	8	9	10	11	12	13	14	15	16	17	18	19	20	total
Fracture type [Mason] after arthroscopy	II	II	IV	IV	II	IV	IV	I	IV	IV	III	IV	IV	IV	IV	IV	IV	II	IV	IV	1:4:1:14
Gender	f	f	m	m	m	f	f	m	f	m	m	m	m	m	m	f	m	m	m	f	f:m = 1:2.9
Age [years]	58	40	39	50	32	50	44	54	44	46	47	28	31	58	31	41	22	59	51	32	42.9 ± 10.9
Follow up [months]	47	46	45	45	44	44	44	43	43	41	41	40	40	40	39	38	37	37	36	35	41.4 ± 3.4
Loose bodies [n]	0	2	4	4	0	1	3	1	1	1	1	2	1	1	1	2	1	0	2	1	85%
Osteochondral lesions capitellum	–	+	+	+	+	–	+	+	+	+	–	+	+	+	+	+	+	–	+	+	80%
Injury to the lateral collateral ligaments	–	–	–	–	–	+	–	–	+	–	–	–	–	+	–	+	+	–	+	+	35%
Screw osteosynthesis	+	+	–	+	–	–	–	–	+	+	+	+	+	–	+	–	+	+	+	+	65%
Partial radial head resection	–	–	+	–	–	–	–	–	–	–	–	–	–	+	–	–	–	–	–	–	10%
Loose body removal	–	+	+	+	–	+	+	+	+	+	+	+	+	+	+	+	+	–	+	+	85%
Chondroplasty capitellum	–	+	+	+	+	–	+	+	+	+	–	–	–	–	–	–	–	–	–	–	40%
Lateral collateral ligament reconstruction	–	–	–	–	–	+	–	–	+	–	–	–	–	+	–	+	+	–	+	+	35%
OES	48	47	47	48	47	48	48	47	48	45	47	48	45	45	48	48	48	48	47	48	47.3 ± 1.1
MEPI	100	95	100	100	100	95	100	100	100	95	100	95	95	95	100	100	100	100	100	100	98.5 ± 2.4
DASH	0	1	0	0	1.7	0	0	0.8	0	1	0	1	2	2	0	0	0	0	1.7	0	0.6 ± 0.8

*f* female, *m* male, *OES* The Oxford Elbow Score, *MEPI* The Mayo Elbow Performance Index, *DASH* Disabilities of Arm, Shoulder and Hand Questionnaire

**Table 2:** Detailed information of patients’ fracture types and associated lesions after conventional radiographs (CR), CT/MRI imaging and elbow arthroscopy (Scope)

		X-Ray	CT/MRI	Scope		X-Ray	CT/MRI	Scope
Classification [Mason]	Patient 1	I	II	II	Patient 11	III	–	III
Fracture fragments [n]		2	3	3		2	–	2
Loose bodies [n]		0	0	0		0	–	1
Capitellar osteo(chondral) lesions		no	no	no		no	–	no
Classification [Mason]	Patient 2	I	II	II	Patient 12	III	–	IV
Fracture fragments [n]		3	3	3		2	–	3
Loose bodies [n]		0	0	2		0	–	2
Capitellar osteo(chondral) lesions		no	no	yes		no	–	yes
Classification [Mason]	Patient 3	I	IV	IV	Patient 13	II	–	IV
Fracture fragments [n]		2	3	3		2	–	2
Loose bodies [n]		0	3	4		1	–	1
Capitellar osteo(chondral) lesions		no	yes	yes		no	–	yes
Classification [Mason]	Patient 4	IV	IV	IV	Patient 14	I	I	IV
Fracture fragments [n]		2	2	2		2	2	2
Loose bodies [n]		0	3	4		1	1	1
Capitellar osteo(chondral) lesions		yes	yes	yes		no	no	yes
Classification [Mason]	Patient 5	II	–	II	Patient 15	II	–	IV
Fracture fragments [n]		2	–	3		1	–	2
Loose bodies [n]		0	–	0		0	–	1
Capitellar osteo(chondral) lesions		no	–	yes		no	–	yes
Classification [Mason]	Patient 6	IV	IV	IV	Patient 16	II	–	IV
Fracture fragments [n]		1	1	1		2	–	2
Loose bodies [n]		1	1	1		0	–	2
Capitellar osteo(chondral) lesions		no	no	no		no	–	yes
Classification [Mason]	Patient 7	I	IV	IV	Patient 17	III	IV	IV
Fracture fragments [n]		2	3	3		3	3	3
Loose bodies [n]		0	1	3		0	0	1
Capitellar osteo(chondral) lesions		no	yes	yes		no	yes	yes
Classification [Mason]	Patient 8	I	I	I	Patient 18	II	–	II
Fracture fragments [n]		2	2	2		1	–	1
Loose bodies [n]		1	1	1		0	–	0
Capitellar osteo(chondral) lesions		no	no	yes		no	no	no
Classification [Mason]	Patient 9	I	–	IV	Patient 19	II	IV	IV
Fracture fragments [n]		1	–	1		2	2	3
Loose bodies [n]		0	–	1		1	1	2
Capitellar osteo(chondral) lesions		no	–	yes		no	yes	yes
Classification [Mason]	Patient 10	III	IV	IV	Patient 20	II	–	IV
Fracture fragments [n]		2	3	3		1	–	1
Loose bodies [n]		0	1	1		0	–	1
Capitellar osteo(chondral) lesions		no	yes	yes		no	–	yes

**Table 3:** Differences in fracture classification, number of fracture fragments, identification of loose bodies and number of loose bodies comparing conventional radiographs (CR) with CT/MRI imaging, CR with elbow arthroscopy as well as CT/MRI imaging with elbow arthroscopy

	Differences in Classification	Differences in number of fracture fragments	Differences in identification of loose bodies	Differences in number of loose bodies
CR -- > Arthroscopy	70%	40%	60%	65%
CR -- > CT/MRI	64%	36%	36%	36%
CT/MRI -- > Scope	9%	9%	18%	55%

During elbow arthroscopy fractures were classified as following: type I in 5%, type II in 20%, type III in 5% and type IV in 70%. Comparing the fracture classification after CR with the arthroscopic findings, the classification changed in 14 cases (70%). Comparing the classification based on CT/MRI imaging and elbow arthroscopy, the classification changed in one case (9%) (Tables 2 and 3).

The changes in classification resulted from 1. the detection of a mechanical block of motion that was not found in the clinical examination, 2. the detection of an anterior rim fragment of the radial head trapped in the posterior compartment, 3. the identification of an varus or posterolateral rotational instability (PLRI) detected during arthroscopy (drive-through-sign) or EUA. In the second and third case the findings revealed an occult dislocation and classification was consequently changed to a type IV injury.

Comparing the number of fracture fragments diagnosed by CR with the findings after CT/MRI and arthroscopy, a higher number of fragments was found in 36 and 40%, respectively (Table 3). In one case arthroscopy revealed an additional fracture fragment that was not diagnosed by the CT scan (Table 3, Fig. 1). Loose joint bodies were diagnosed in 25% of all CR and in 85% during all elbow arthroscopies. The examinations by CT/MRI revealed loose bodies that had not been detected by CR in 36% (4 of 11 cases). In one case arthroscopy revealed loose bodies that were not diagnosed in the CT examination. Analyzing the cases, where loose bodies were identified, CT/MRI examination and arthroscopy revealed a higher number of loose bodies in 50 and 76% when compared to CR, respectively. In 60% additional loose bodies could be diagnosed during elbow arthroscopy that where not detectable with CT/MRI imaging. (Osteo-)chondral lesions of the capitellum humeri were found in 80% during arthroscopic evaluation. These lesions were missed by CR in 94% (15 of 16 cases) and by CT/MRI in 27% (3 of 11 cases). Injuries to the lateral collateral ligament complex were diagnosed during surgery in 35%. In the cases where MRI was performed, all of these injuries were identified prior to arthroscopy.

During arthroscopy percutaneous screw osteosynthesis was performed in 65%, a partial radial head resection in 10%, chondroplastic or microfracturing at the capitellum humeri or the radial head in 85% and a mini-open collateral ligament reconstruction at the humeral insertion was carried out in 35% using suture anchors (3.5 mm Bio Corkscrew or 2.9 mm Suture Tak, Arthrex, USA).

The final clinical outcome assessments resulted in an OES 47.3 ± 1.1, a MEPI of 98.5 ± 2.4 and a DASH score of 0.6 ± 0.8.

## Discussion

To date, the evidence concerning arthroscopically assisted treatment of radial head fractures is limited. There are only a few small case-series available that mainly focus on the feasibility of arthroscopic procedures. The present study represents the largest case series of arthroscopically assisted radial head fracture fixation and, for the first time, it compares findings of preoperative imaging versus arthroscopy with special regards to associated lesions.

In clinical practice conventional radiographs (CR) represent the gold standard of radiologic examination and provide an essential element for radial head fracture classification [27]. Nevertheless, the sensitivity of plain radiographs has been shown to be as low as 21%, at least for simple elbow fractures in a cadaver study [28]. The radiocapitellar view, as performed in our study, can additionally detect radial fractures in up to 5% of patients with no fracture seen in the two standard planes [29]. However, the interobserver reliability of the modified mason classification is poor to moderate (k = 0.45–0.85) and observers likely disagree about the grade of displacement (Mason I vs. II) in plain radiographs [27]. In doubt, some authors conclude that CT or MRI studies should be conducted in addition. In our study, CT or MRI was conducted whenever the extend of dislocation was not assessable on CR, associated lesion were suspected or in case of discrepancy between CR findings and clinical examination. Information gained by CT/MRI imaging led to a change of fracture classification in 64%. Haapamaki et al. investigated 56 patients a with blunt elbow trauma and found that CT revealed 13 fractures that had been missed by plain x-ray study [30]. Acar et al. demonstrated that CT revealed fractures in 12.8% of patients, with positive elbow extension test and normal x-ray study [31]. In terms of interobserver reliability CT examination revealed better results than CR concerning radial head classification [32]. The clinical examination represents another essential element in the Hotchkiss modified Mason classification. The mechanical block of motion is a crucial parameter that per definition is not present in Mason I fractures. Furthermore, it indicates a relevant dislocation or an associated lesions such as loose bodies even if not detected in plain radiographs. Unfortunately, clinical examination might be limited due to unspecific symptoms, such as pain, swelling and joint effusion. In our study, elbow arthroscopy including examination under anesthesia and full visualization of the radio-capitellar and radio-ulnar articulation led to a change of fracture classification in 70% when compared to CR. When comparing the fracture classification after CT/MRI to the classification after arthroscopy, we only revealed a discrepancy in one case. Taken together, arthroscopy does not seem to substantially contribute to a better fracture classification when compared to CT/MRI. Given the limitations of plain radiographs in terms of accurate fracture classification, we recommend CT or MRI scans in cases where clinical examination is hindered and CR does not provide accurate visualization of fracture pattern or fragment dislocation. Hotchkiss et al. also recommended CT scans for additional information on fracture fragment size and displacement [9].

In contrast, arthroscopy revealed superior sensitivity for identifying and quantifying loose joint bodies compared to CT/MRI. While 60% of the loose joint bodies found during arthroscopy were missed in CR, the vast majority was detected by CT/MRI imaging. However, in 60% percent of the cases arthroscopy revealed a larger number of loose bodies than described in CT/MRI (Table 3). These advantages might result from an inappropriate slice thickness of standard CT and MRI scans or low sensitivity in detecting chondral flake fractures. In our study we found loose bodies in 85%. Respecting the fact that osteochondral lesion of the capitellum were found in 80% of the cases, the loose joint bodies not only originated from the radial head fracture, but also from capitellar lesions. Furthermore, injuries to the lateral collateral ligaments became evident during examination under anesthesia and arthroscopy in 35%. Loose joint bodies, osteochondral lesions of the humeral capitellum and lesions to the collateral ligaments are known to be common injuries associated with radial head fractures [10, 12, 33, 34]. Our findings go well in line with Itamura et al. who revealed loose bodies in 22 of 24 (92%) MRIs of radial head fractures [35]. Ward et al. found an incidence of 24% capitellar lesions during open surgery on radial head fractures [36]. Michels et al. found 14% capitellar cartilage lesions during arthroscopic treatment of type II fractures [20]. In our study, (oseto-)chondral lesions to the capitellum were identified in a higher number, which might be due to the high incidence of type IV fractures. Combinations of fractures to the radial head and corresponding capitellar lesions might particularly affect the outcome since 60% of the axial load at the elbow is transmitted through the radiocapitellar joint [37]. Caputo et al. published a case series of capitellar chondral lesions that have been trapped between the fracture fragments of radial head fractures [36]. They also stressed the importance of complete removal of loose joint bodies. Van Riet et al. reported on less good results in the patients with lesions of the capitellum [34] and recommended fixation of larger displaced fractures and excision of small fragments. According to these recommendations we conducted removal of loose bodies in 85% and a chondroplasty in 40%. In our series none of the (osteo-)chondral fragments was suitable for refixation, nevertheless we performed microfracturing in one case of a larger chondral shear lesion (Fig. 1). Unfortunately, there is a lack of literature on the management of traumatic cartilage lesions to the capitellum and evidence-based recommendations are missing.

In our cohort, injuries to the lateral collateral ligament complex (LCL) were found in 35%. This incidence seems remarkably high. However, other studies demonstrated that the incidence of LCL injuries increases with the severity of in radial head fractures [34, 38, 39]. Again, the high percentage of type IV fractures in our study may account for the high incidence of LCL injuries. On the other hand, the routinely conducted combination of EUA and diagnostic arthroscopy may allow for a higher sensitivity for detection of PLRI. Elbow arthroscopy has been shown to be a valuable tool in diagnosing and management of elbow instability, such as PLRI [16, 40]. Holt et al. suggested the anteromedial portal may be used while performing a pivot shift maneuver. If PLRI is present, the radial head may be seen rotating and translating posteriorly during this maneuver [40]. In our study, we used a modified “elbow drive-through sign” which originally is performed with the arthroscope in the anterolateral portal. In patients with PLRI, the arthroscope is easily driven through the lateral gutter and into the lateral aspect of the ulnohumeral joint [15, 23, 41]. In our modified technique the switching stick from was introduced through the soft spot portal with the arthroscope in the anterolateral portal. When the switching stick can easily be advanced between ulnar and humerus towards the coronoid process PLRI is present (Fig. 3). Using this technique, injuries to the cartilage by the arthroscope itself can be minimized. Given the importance of LCL complex restoration [16], we conducted a mini-open repair of the LCL (mainly LUCL) whenever PLRI was diagnosed.

Apart from the accompanying injuries, the actual radial head fracture was treated by arthroscopically assisted percutaneous screw osteosynthesis in 65% and by partial radial head resection in 10%. The partial radial head resection was conducted in cases of small-sized and shallow anterior rim fractures that were not suitable for refixation. Rolla et al. first described a standard approach for arthroscopic fixation of radial head fractures with cannulated differential thread screws in a case-series of six patients. The authors particularly pointed out the benefits of simultaneous treatment of associated lesions, such as chondral avulsion of the capitellum. The authors found satisfactory short-term preliminary outcomes [21]. Michels et al. [20] retrospectively evaluated the results of arthroscopically assisted reduction and percutaneous fixation of radial head fractures in 14 patients. The authors reported on eleven excellent and three good results in their study population consisting of Mason II fractures only. In contrast, our study focused on radial head fractures with evidence of associated injuries. Therefore, in our study population type III and IV fractures were diagnosed in 75%. Despite this high ratio of severe injuries we found excellent outcome scores in all cases after a mid-term follow up of 41.4 ± 3.4 months. Comparing these results to the outcomes after open reduction and internal fixation [42, 43], arthroscopically assisted fracture fixation led to similar or slightly superior results.

This study has its limitation due to a retrospective design, a lack of a control group and an incomplete dataset of CT or MRI examination. Furthermore, we must emphasize that arthroscopic radial head fracture reduction and fixation is a technically demanding procedure. We do not consider this procedure as a standard of care in general trauma service, since it requires the skills of experienced arthroscopists. However, a valuable point of arthroscopy in diagnosing associated injuries is the possibility to avoid treatment delay. MRI is costly which later can affect patient decision that will delay the treatment.

## Conclusions

Elbow arthroscopy has a significant diagnostic value in radial head fractures. Our study demonstrates that elbow arthroscopy features a high accuracy of fracture classification and identification of relevant associated injuries, such as loose bodies and capitellar lesions. Moreover, all intra-articular lesions can be treated arthroscopically. Arthroscopically assisted fracture reduction and internal fixation reduces invasiveness and reliably allows for excellent clinical outcomes.

## Data Availability

All data generated or analysed during this study are included in this published article.

## Abbreviations

C: Capitellum
CP: Coronoid process
CR: Conventional radiographs
CT: Computed tomography
DASH: Disabilities of Arm, Shoulder and Hand Questionnaire
EUA: Examination under anesthesia
f: Female
H: Humerus
LB: Loose bodies
LCL: Lateral collateral ligament complex
m: Male
MEPI: Mayo Elbow Performance Index
MRI: Magnetic resonance imaging
OES: Oxford Elbow Score
PLRI: Posterolateral rotational instability
PRUJ: Proximal radioulnar joint
RH: Radial head
U: Ulna

## References

[1] Wang ML, Beredjiklian PK (2015). Management of radial head fracture with elbow dislocation. J Hand Surg Am.

[2] Gao Y, Zhang W, Duan X, et al. Surgical interventions for treating radial head fractures in adults. Cochrane Database Syst Rev. 2013;(5):CD008987. 10.1002/14651858.CD008987.pub2 PMC12145950 23728684

[3] Shulman BS, Lee JH, Liporace FA, Egol KA (2015). Minimally displaced radial head/neck fractures (Mason type-I, OTA types 21A2.2 and 21B2.1): are we "over treating" our patients?. J Orthop Trauma.

[4] Ruchelsman DE, Christoforou D, Jupiter JB (2013). Fractures of the radial head and neck. J Bone Joint Surg Am.

[5] Yeoh KM, King GJ, Faber KJ, Glazebrook MA, Athwal GS (2012). Evidence-based indications for elbow arthroscopy. Arthroscopy..

[6] Crosby NE, Greenberg JA (2014). Radiographic evaluation of the elbow. J Hand Surg Am..

[7] Guitton TG, Brouwer K, Lindenhovius AL (2014). Diagnostic accuracy of two-dimensional and three-dimensional imaging and modeling of radial head fractures. J Hand Microsurg.

[8] Mason ML (1954). Some observations on fractures of the head of the radius with a review of one hundred cases. Br J Surg.

[9] Hotchkiss RN (1997). Displaced fractures of the radial head: internal fixation or excision?. J Am Acad Orthop Surg.

[10] Smits AJ, Giannakopoulos GF, Zuidema WP. Long-term results and treatment modalities of conservatively treated Broberg-Morrey type 1 radial head fractures. Injury. Br J Surg. 1954;42(172):123–32. 10.1016/j.injury.2014.05.034 24975654

[11] Yoon A, King GJ, Grewal R (2014). Is ORIF superior to nonoperative treatment in isolated displaced partial articular fractures of the radial head?. Clin Orthop Relat Res.

[12] Johnston GW (1962). A follow-up of one hundred cases of fracture of the head of the radius with a review of the literature. Ulster Med J.

[13] Vester H, Siebenlist S, Imhoff AB, Lenich A (2014). Arthroscopy of the elbow: diagnostic and therapeutic approaches. Orthopäde..

[14] Hsu JW, Gould JL, Fonseca-Sabune H, Hausman MH (2009). The emerging role of elbow arthroscopy in chronic use injuries and fracture care. Hand Clin.

[15] Savoie FH, Field LD, Gurley DJ (2009). Arthroscopic and open radial ulnohumeral ligament reconstruction for posterolateral rotatory instability of the elbow. Hand Clin.

[16] Goodwin D, Dynin M, Macdonnell JR, Kessler MW (2013). The role of arthroscopy in chronic elbow instability. Arthroscopy..

[17] Atesok K, Doral MN, Whipple T (2011). Arthroscopy-assisted fracture fixation. Knee Surg Sports Traumatol Arthrosc.

[18] Adams JE, Merten SM, Steinmann SP (2007). Arthroscopic-assisted treatment of coronoid fractures. Arthroscopy..

[19] Fink Barnes LA, Parsons BO, Hausman M (2015). Arthroscopic Management of Elbow Fractures. Hand Clin.

[20] Michels F, Pouliart N, Handelberg F (2007). Arthroscopic management of Mason type 2 radial head fractures. Knee Surg Sports Traumatol Arthrosc.

[21] Rolla PR, Surace MF, Bini A, Pilato G (2006). Arthroscopic treatment of fractures of the radial head. Arthroscopy.

[22] Wijeratna M, Bailey KA, Pace A, Tytherleigh-Strong G, Van Rensburg L, Kent M (2012). Arthroscopic radial head excision in managing elbow trauma. Int Orthop.

[23] Savoie FH, O'Brien MJ, Field LD, Gurley DJ (2010). Arthroscopic and open radial ulnohumeral ligament reconstruction for posterolateral rotatory instability of the elbow. Clin Sports Med.

[24] Hudak PL, Amadio PC, Bombardier C (1996). Development of an upper extremity outcome measure: the DASH (disabilities of the arm, shoulder and hand) [corrected]. The upper extremity collaborative group (UECG). Am J Ind Med.

[25] Dawson J, Doll H, Boller I (2008). The development and validation of a patient-reported questionnaire to assess outcomes of elbow surgery. J Bone Joint Surg Br.

[26] An KN, Morrey BF. Functional evaluation of the elbow. 2nd ed. Philadelphia: WB Saunders; 1993.

[27] de Muinck Keizer RJ, Walenkamp MM, Goslings JC, Schep NW (2015). Mason type I fractures of the radial head. Orthopedics..

[28] McGinley JC, Roach N, Hopgood BC, Kozin SH (2006). Nondisplaced elbow fractures: a commonly occurring and difficult diagnosis. Am J Emerg Med.

[29] Hall-Craggs MA, Shorvon PJ, Chapman M (1985). Assessment of the radial head-capitellum view and the dorsal fat-pad sign in acute elbow trauma. AJR Am J Roentgenol.

[30] Haapamaki VV, Kiuru MJ, Koskinen SK (2004). Multidetector computed tomography diagnosis of adult elbow fractures. Acta Radiol.

[31] Acar K, Aksay E, Oray D, Imamoglu T, Gunay E (2016). Utility of computed tomography in elbow trauma patients with Normal X-ray study and positive elbow extension test. J Emerg Med.

[32] Guitton TG, Ring D (2011). Science of variation G. Interobserver reliability of radial head fracture classification: two-dimensional compared with three-dimensional CT. J Bone Joint Surg Am.

[33] Jeon IH, Micic ID, Yamamoto N, Morrey BF (2008). Osborne-cotterill lesion: an osseous defect of the capitellum associated with instability of the elbow. AJR Am J Roentgenol.

[34] van Riet RP, Morrey BF (2008). Documentation of associated injuries occurring with radial head fracture. Clin Orthop Relat Res.

[35] Itamura J, Roidis N, Mirzayan R, Vaishnav S, Learch T, Shean C (2005). Radial head fractures: MRI evaluation of associated injuries. J Shoulder Elb Surg.

[36] Ward WG, Nunley JA (1988). Concomitant fractures of the capitellum and radial head. J Orthop Trauma.

[37] Morrey BF, An KN, Stormont TJ (1988). Force transmission through the radial head. J Bone Joint Surg Am.

[38] Johansson O. Capsular and ligament injuries of the elbow joint. A clinical and arthrographic study. Acta Chir Scand Suppl. 1962;(Suppl 287):1–159. 14451962

[39] Kaas L, van Riet RP, Turkenburg JL, Vroemen JP, van Dijk CN, Eygendaal D (2011). Magnetic resonance imaging in radial head fractures: most associated injuries are not clinically relevant. J Shoulder Elb Surg.

[40] Holt MS, Savoie FH, Field LD, Ramsey JR (2004). Arthroscopic management of elbow trauma. Hand Clin.

[41] Cheung EV (2008). Chronic lateral elbow instability. Orthop Clin North Am.

[42] Ring D, Quintero J, Jupiter JB (2002). Open reduction and internal fixation of fractures of the radial head. J Bone Joint Surg Am.

[43] Bruinsma W, Kodde I, de Muinck Keizer RJ (2014). A randomized controlled trial of nonoperative treatment versus open reduction and internal fixation for stable, displaced, partial articular fractures of the radial head: the RAMBO trial. BMC Musculoskelet Disord.

